# Modeling the impact of universal TB molecular testing and timing of TB preventive treatment during ART initiation in South Africa

**DOI:** 10.1097/QAD.0000000000003707

**Published:** 2023-08-30

**Authors:** Ruchita BALASUBRAMANIAN, Kate SHEARER, Don MUDZENGI, Piotr HIPPNER, Jonathan E. GOLUB, Violet CHIHOTA, Christopher J HOFFMANN, Emily A KENDALL

**Affiliations:** 1 Harvard T.H. Chan School of Public Health, Boston, MA, USA; 2 Division of Infectious Diseases, Johns Hopkins University School of Medicine, Baltimore, MD, USA; 3 Aurum Institute, Johannesburg, South Africa; 4 University of Witwatersrand, Johannesburg, South Africa; 5 Division of Infectious Diseases, Department of Medicine, Vanderbilt University, Nashville, USA

**Keywords:** Prevention, Early diagnosis, Tuberculosis, HIV, South Africa

## Abstract

**Objectives:**

Targeted universal tuberculosis (TB) testing can improve TB detection among people with HIV. This approach is being scaled up in South Africa through Xpert MTB/RIF Ultra testing for individuals starting antiretroviral therapy and annually thereafter. Clarity is needed on how Universal Xpert testing may affect TB preventive treatment (TPT) provision, and on whether TPT should be delayed until TB is ruled out.

**Design:**

State-transition microsimulation.

**Methods:**

We simulated a cohort of South African patients being screened for TB while entering HIV care. We compared clinical and cost outcomes between four TB screening algorithms: symptom-based, C-reactive protein-based, and Universal Xpert testing with either simultaneous or delayed TPT initiation.

**Results:**

Prompt TB treatment initiation among simulated patients with TB increased from 26% (24%–28%) under symptom screening to 53% (50–56%) with Universal Xpert testing. Universal Xpert testing led to increased TPT uptake when TPT initiation was simultaneous, but to approximately 50% lower TPT uptake if TPT was delayed. Universal Xpert with simultaneous TPT prevented incident TB compared to either symptom screening (median 17 cases averted per 5000 patients) or Universal Xpert with delayed TPT (median 23 averted). Universal Xpert with Simultaneous TPT cost approximately $39 per incremental TPT course compared to Universal Xpert with delayed TPT.

**Conclusions:**

Universal Xpert testing can promote timely treatment for newly diagnosed people with HIV who have active TB. Pairing universal testing with immediate TPT will improve the promptness, uptake, and preventive effects of TPT. Simultaneous improvements to TB care cascades are needed to maximize impact.

## Introduction

South Africa is among the countries with the highest incidence of both HIV and tuberculosis (TB).[1] There are an estimated 7.5 million people living with HIV (PWH) in South Africa, and TB is their leading cause of death.[2,3] The scale-up of antiretroviral therapy (ART) has lowered the incidence of TB among PLHIV due to the protective effect of immune recovery against TB risk.[4] Achieving further reductions in TB morbidity, mortality, and transmission among PLHIV requires further innovation in case detection and preventive approaches.

Enhanced case detection and TB preventive treatment (TPT) can improve TB control among PLHIV. Multiple randomized and observational studies have reported improved outcomes among PLHIV who receive TPT during ART initiation, including reduced TB incidence and reduced mortality.[5,6] Additionally, modeling studies have estimated the public health impact and cost-effectiveness of further scale-up of TPT. [7] Thus, TPT for PLHIV is strongly recommended in international guidelines from the World Health Organization (WHO).[8] South Africa has been a global leader in scaling up TPT for PLHIV, yet provision remains below the National Strategic Plan’s goal of reducing TB transmission by increasing the uptake of TPT to those that are eligible, with 63% of those who initiated ART receiving TPT.[9–11] Ruling out active TB impedes prescribing TPT, and studies of the impact of TPT do not provide direct guidance about how to screen for active TB when initiating TPT. The WHO recommends a four-symptom TB screen (chronic cough, fever, weight loss and night sweats) during ART initiation, followed by confirmatory microbiologic testing for those with symptoms.[12–14] However, symptom screening has limited sensitivity (83%) and poor specificity (38%).[15]

South Africa’s post-COVID TB recovery plan recommends targeted universal Xpert Ultra testing for all PLHIV regardless of symptoms.[16] This approach is aimed at increasing the detection of subclinical TB, and is likely to increase the universality of TB testing among symptomatic individuals.[17] In addition to increasing TB detection, universal molecular testing among people entering HIV care can increase TPT provision by reassuring clinicians that active TB has been excluded. However, current implementations of this strategy defer TPT and ART initiation to a later visit when TB results are available. This has two limitations: (1) Even if Xpert results are reported to the clinician within days, TPT initiation will be delayed, possibly for weeks, until a patient returns to the clinic, and (2) TPT may be neglected if it is not reconsidered during the workflow of follow-up visits. As a result, overall TPT provision may decline, and its onset will be delayed during the early-ART period when TB incidence and mortality are highest and the potential impact of TPT is greatest.[5,6,18]

Alternative approaches include initiating TPT simultaneously with universal Xpert testing. Once Xpert results are available, patients with positive results would switch to TB treatment. This approach would briefly expose many patients with active TB to one/two-drug TPT, but it could ultimately improve outcomes by simultaneously increasing TB detection and early TPT delivery. To understand the expected benefits and costs of a universal TPT initiation strategy relative to conventional and evolving standards of care, and to explore the dependence of these outcomes on care cascade implementation, we designed a microsimulation of TB diagnosis and TPT initiation among a cohort of ambulatory PLHIV entering HIV care and initiating ART in South Africa.

## Methods

We constructed a state-transition micro-simulation model of adult PLHIV evaluated for ART initiation in a South African ambulatory setting. We limit the model to individuals initiating ART because they have the clearest opportunity to enter TB preventive care pathways and are the primary focus of new guidelines for TB testing among PLHIV in South Africa.[19] Focusing on new HIV patients eligible for TB treatment or TPT in ambulatory care settings, we modeled patients without danger signs requiring hospitalization and liver disease that would preclude standard TB therapy. We excluded patients for whom an initial TB screening step does not occur or who do not return within 90 days of the first visit.

**Figure 1.**
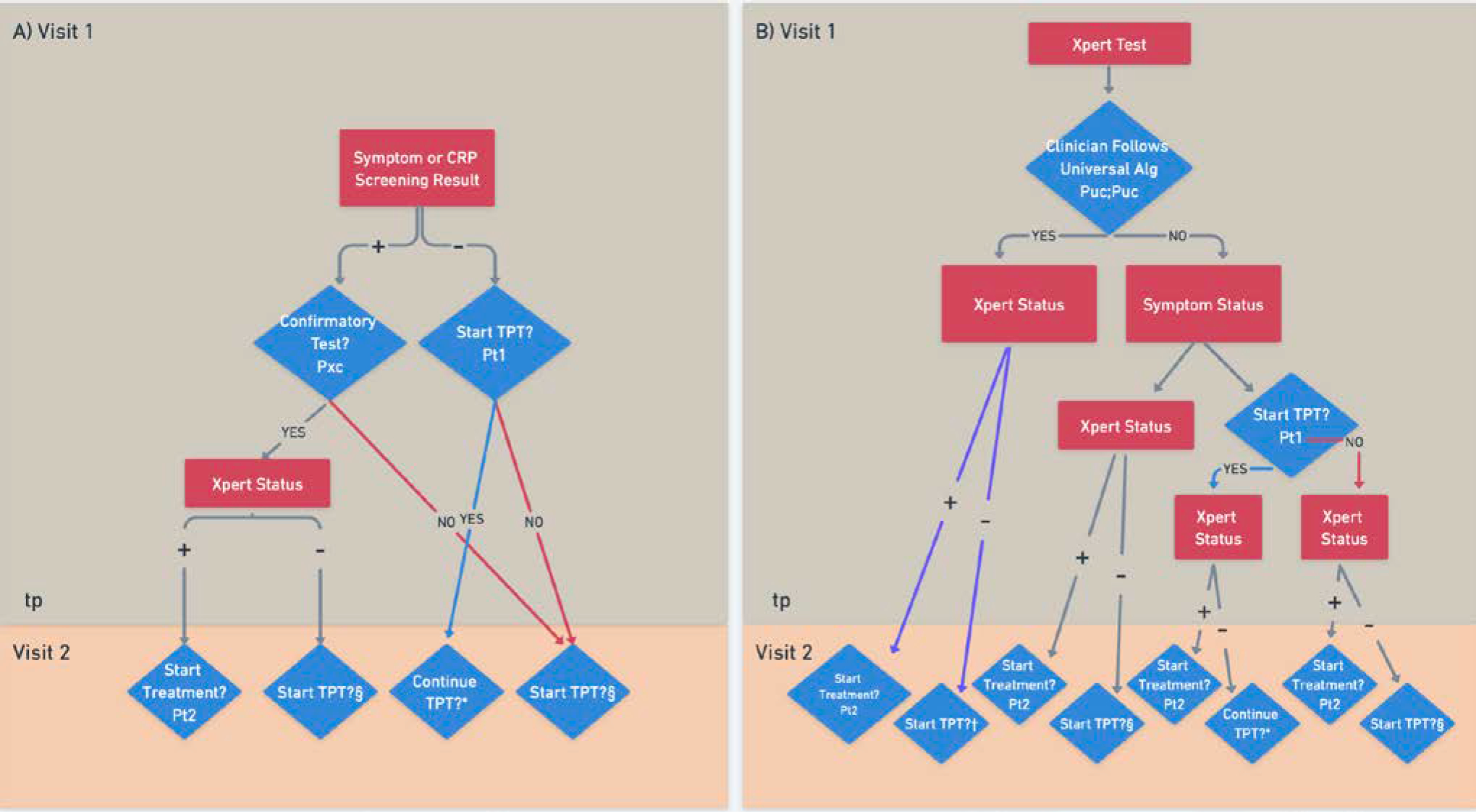
Schematic of Screening Algorithms. Blue diamonds represent probabilities of completing steps in the care cascade. Red rectangles represent patient status on tests that may be performed, as assigned to the cohort in a TB-status- and CD4-dependent manner prior. **A)** Schematic for Symptom Screening and CRP Screening Algorithms **B)** Schematic for Universal Xpert with Delayed TPT and Universal Xpert with Simultaneous TPT Algorithms. * TPT continues with probability 1−d_t_ if the follow-up visit occurs within 31 days, and with probability P_31T_ if beyond 31 days. §TPT is started with probability RR_T2_ P_T1_. †In the Universal Xpert with Simultaneous TPT Algorithm, TPT continues with probabilities as in *; in the Universal Xpert with Delayed TPT Algorithm, TPT is started with probability RR_T2_ P_T1_. Blue lines mean that TPT was initiated, red lines mean that TPT was not initiated, and purple lines mean that TPT was either initiated or delayed depending on the algorithm.

We modeled patients’ baseline CD4 count and active TB status; their initial HIV visit including any TB screening, diagnostic testing, and/or TPT initiation; their first follow-up visit and any TPT or TB treatment provided at that visit; and, for those who did not have active TB at baseline, their TB incidence over the subsequent two years. We estimate expected differences in TPT initiation, TB treatment initiation, TB incidence, resulting mortality, and health system cost between four different screening algorithms (Figure 1):

1. Symptom screening with confirmatory Xpert MTB/RIF Ultra testing, representing traditional approaches in South Africa,
2. C-reactive protein (CRP) as a TB screening test, with confirmatory Xpert testing,
3. Universal Xpert screening with delayed TPT initiation
4. Universal Xpert screening with simultaneous TPT initiation

We generated a 5000-person cohort and simulated its outcomes under each of the four TB-screening algorithms. To represent uncertainty in clinical, cost, and cost-effectiveness outcomes, 1000 independent sets of data-consistent parameter values were sampled from triangular distributions based on existing literature or ongoing studies (Table S1), and results are presented as a median and interquartile range across the 1000 simulated cohorts (Methods S1, S2). To confirm the adequacy of our cohort size, we compared simulated clinical outcomes across 5 independent runs (Table S8). Descriptions of care cascades are reported as a mean proportion completing each step.

### Baseline Characteristics

Each patient was assigned an initial CD4 count stratum (<100, 100–200, 200–350, >350), active pulmonary TB status (TB or no TB), symptom-screen status (positive or negative), CRP status (abnormal [≥10 mg/L] or normal [<10 mg/L]), and Xpert status (positive, negative, or unable to produce sputum), using parameter values reflective of PLHIV in South Africa (Table S1). Timing of the first follow-up visit and expected adherence to ART and TPT, if prescribed, were also determined at baseline for each cohort and held constant across all TB screening algorithms.

### TB Assessment and TPT Algorithm

We explicitly modeled the TB diagnostic or preventive interventions provided at the initial clinic visit and first follow-up visit under each screening algorithm (Figure 1;Table S1). Care cascades were estimated empirically from pragmatic clinical trials in South Africa, for an active TB cascade (with steps of receiving a TB test at the first visit, having a positive Xpert test, and starting treatment at the first follow-up visit) and a TPT cascade (with steps of starting TPT at the initial visit, starting or continuing TPT at the first follow-up visit, completing ≥6 months of TPT, and completing ≥12 months of TPT).

In the Symptom and CRP Screening algorithms, patients receive a symptom screen or a point-of-care CRP test, respectively, at the initial visit. Patients who screen positive are offered a confirmatory sputum Xpert test with probability P_XC_ (<1 due to gaps in the care continuum). The next visit occurs at a median of 28 days later. Those who screen negative initiate TPT with probability P_T1_.

In the Universal Xpert algorithms, all patients entering HIV care are offered a sputum Xpert test. We assume that some clinicians may be hesitant to universally start or, to a lesser extent, universally delay TPT while awaiting Xpert results; thus, for a fraction of patients (1−P_UC_/1−P_UD_, respectively;Table S1), the TPT initiation decision is based on the result of the same clinician-performed TB symptom screen as in the Symptom Screening algorithm. For remaining positive Xpert results, clinicians who see patients at their follow-up visits may not be aware of or act on their results, so TB goes untreated. For the rest of the cohort, TPT is either delayed (Universal Xpert with Delayed TPT) or prescribed for 30 days (Universal Xpert with Simultaneous TPT) while awaiting Xpert results, regardless of symptoms.

At patients’ first follow-up visit, those with a positive Xpert result (including trace results, which indicate detection of low quantities of M. tuberculosis DNA) initiate TB treatment with probability P_T2_; this includes those who had previously initiated TPT. For remaining positive Xpert results (1−P_T2_), clinicians who see patients at their follow-up visits may not be aware of or act on their Xpert results, so TB goes untreated. Those eligible for TPT initiate it with probability P_T1_*RR_T2_ (a reduction relative to the initial visit, reflecting non-routine TPT initiation at follow-up visits).

TPT is modeled as a 12-month oral regimen (per current South African guidelines for isoniazid TPT), and we assume that patients must complete ≥6 months to benefit from a long-term protective effect after it is discontinued. Those whose first follow-up visit occurs >31 days after TPT initiation have a probability 1−P_31T_ (10–40%, Table S1) of not resuming TPT after the initial treatment gap. Beyond that first follow-up visit, TPT is subject to early discontinuation at a monthly rate d_t_ of 4–10% (Table S1), inclusive of TPT discontinuations by patients who also discontinue ART and/or are lost to follow up from HIV care.

To focus on the effects of TB-specific interventions, ART initiation is modeled similarly under all algorithms and uses the result of TB screening with either CRP (CRP algorithm) or a symptom screen (remaining algorithms). ART is started at the initial visit for all who screen negative for TB by this this initial screening test and for 50% of those who screen positive. For patients who did not start ART immediately, the protective effects of ART begin after their first follow-up visit. Once started, ART continues indefinitely, subject to a monthly loss to follow-up rate d_a_ (1–5%;Table S1) those who discontinue ART are assumed to also discontinue TPT.

### TB Incidence and Mortality Model

Those without TB at baseline are followed for two years to estimate incident TB. TB incidence rates are time-varying and depend on baseline CD4 count, ART status (not on ART, on ART<6 months, or on ART>6 months), and TPT status (currently on TPT, not on TPT but completed ≥180 days of TPT, or not on TPT and never completed 180 days of TPT) (Table S2;S3). Any TB treatment given to a patient without active TB is assumed to protect against progression of latent TB infections to the same degree as TPT.

We used long-term clinical trial follow-up data to estimate the number of deaths averted/incident TB case prevented by TPT to estimate differences in TB mortality between algorithms only among those with no active TB at baseline.[5]

### Costs

We estimated the following costs to the health system, in 2022 USD (all costs are reported here and in Methods S1):

- Xpert testing at the initial visit, estimated at $33.50 per test[20]
- CRP screening/person at the initial visit, estimated at $2.50[21,22]
- Isoniazid preventive treatment administration, estimated as a monthly unit cost of $2.84 applied to the number of months prescribed
- TB treatment, estimated as an average cost per treatment course initiated ($130)[23–25]

### Sensitivity Analysis

We examined sensitivity of two outcomes to individual model parameters: the difference in incident TB, comparing Simultaneous versus Delayed TPT (each with universal Xpert); and the difference in treatment initiation among patients with active TB at baseline, comparing Universal Xpert with Simultaneous TPT versus Symptom Screening. For each parameter, we compared the distribution of outcome values between simulations in which the parameter took the upper quintile values and those in which the same parameter took the lowest quintile values (Figure S1). See Methods S3 for more details.

The following scenarios were also considered:

1. Reducing follow-up visit completion from 100% to a more typical 75% (Table S4),
2. Concentrating the risk of incident TB shortly after initiation of ART (by increasing incidence rates three-fold during the first two months, and decreasing it thereafter to maintain a constant one-year cumulative incidence in absence of TPT;Table S5),
3. Assuming no TPT discontinuation once initiated (Table S6).

## Results

**Figure 2.**
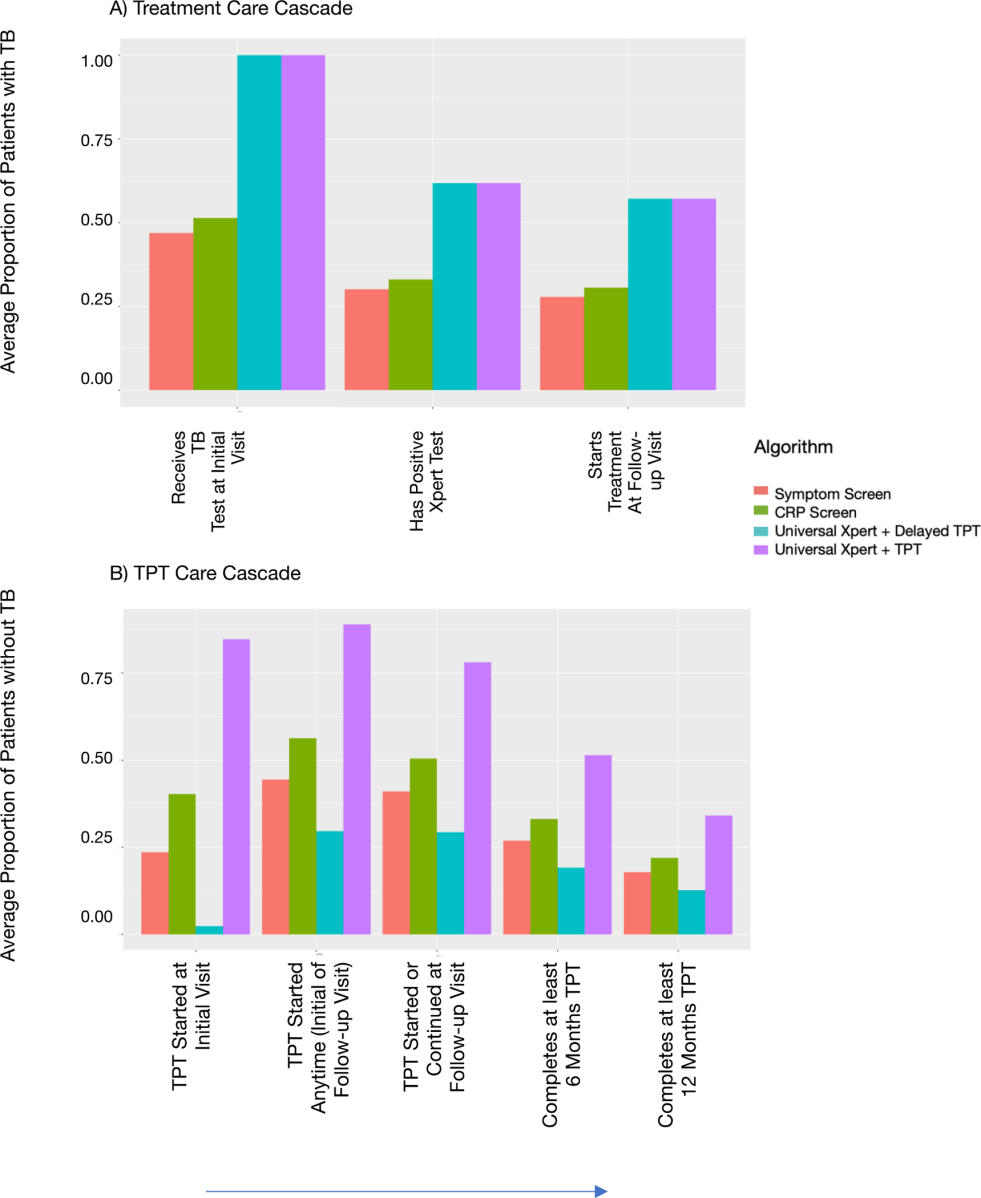
Treatment and TPT Cascades of Care. Panel A shows completion of steps leading to treatment, as a proportion of those patients who have TB, and Panel B shows completion of steps leading to TPT completion, among patients who do not have TB. Bars heights indicate the mean proportion of patients with TB (panel A) or without TB (panel B) who complete each step, across 1000 simulations of the cohort for the indicated screening algorithm. The X-axis represents progression along the treatment cascade of care (panel A) and the TPT cascade of care (panel B).

In a hypothetical cohort of 5000 patients initiating ART, Universal Xpert testing (whether accompanied by Delayed or Simultaneous TPT) resulted in an approximate two-fold increase in TB treatment initiation at the first follow-up visit. Among the 10% (9–11%) of the cohort with TB, 26% (24–28%) were initiated on TB treatment with symptom screening alone, 28% (26–31%) with CRP screening, and 53% (50–56%) with either Universal Xpert algorithm (Figure 2A;Table 1). Under either Universal Xpert algorithm, treatment coverage was limited mainly by Xpert’s sensitivity (Figure 2A) for population-based screening. Adding typical levels of follow-up visit noncompletion to the model (25%) further limited TB treatment completion to only 37% (35–39%) of patients with TB (Table S4). Universal Xpert increased TB treatment initiation among individuals without sputum-culture-positive TB (due to imperfect specificity), such that up to a third of positive Xpert tests were potential false-positives (Table 1).

When adopting Universal Xpert testing, TPT initiation differed substantially between the delayed versus simultaneous TPT algorithms (Table 1). Of patients without TB, 88% (86–90%) initiated TPT within 60 days under Universal Xpert with Simultaneous TPT, versus only 23% (17–31%) under Universal Xpert with Delayed TPT. Universal Xpert with Simultaneous TPT also resulted in more sustained continuation of TPT beyond the follow-up visit (median 72% of patients without TB, compared to 38% for Symptom Screening and 48% for CRP Screening). With the considerable attrition modeled under all algorithms, only 59–65% of those who started TPT remained on TPT for ≥6 months. Universal Xpert with Simultaneous TPT maximized the proportion of patients with TB receiving continued TPT (Table 1): 32% (29–34%) compared to 22%, 19%, and 8% under Symptom Screening, CRP, and Delayed TPT algorithms, respectively. Most of the patients with TB who continued TPT in the Universal Xpert with Simultaneous TPT algorithm had false-negative Xpert testing and, thus, likely a lower bacillary burden.

Among those without TB at ART initiation, incident TB ranged from 3.4–3.8% across algorithms (Table 1). Universal Xpert with Simultaneous TPT prevented incident TB (and consequently, TB mortality) compared to the alternatives: a median of 17 cases averted relative to Symptom Screening, and 23 cases averted relative to Universal Xpert with Delayed TPT (Table 2). In sensitivity analyses that more heavily concentrated TB risk within the first 2 months after ART initiation, this impact increased to median 27 and 37 incident TB cases averted, respectively (Table S5). Indicative of the strong protective effect of ART, half of the incident TB, regardless of algorithm, occurred among patients who had disengaged from care and ART. In a sensitivity analysis assuming 100% continuity of ART and 100% completion of initiated TPT, incident TB decreased to 2.0–2.3% (Table S6)

Universal Xpert testing increased health system costs, primarily due to increased case detection and TB treatment and also the cost of Xpert testing itself (Table S7). Compared to Universal Xpert with Delayed TPT, the Simultaneous TPT alternative cost $39/incremental TPT course initiated, $2,492/incident TB case prevented, and $3,738/death averted (Table 3).

In sensitivity analyses, the number of incident TB cases averted by Simultaneous (versus Delayed) TPT was most sensitive to the probability of initiating TPT at a follow-up visit (Figure S1A). The impact of Universal Xpert (with Simultaneous TPT, relative to Symptom Screening) on treatment initiation was most affected by the probability of administering an Xpert test following a positive initial screen and the TB prevalence in the cohort (Figure S1B).

## Discussion

For a simulated cohort of 5000 people initiating ART, the replacement of symptom-based TB screening with Universal Xpert testing led to a two-fold increase in the proportion of patients with TB who started TB treatment within 60 days. When TPT was started simultaneously with Universal Xpert, TPT delivery doubled compared to symptom screening. Thus, based on current patterns of service delivery, our analysis supports Universal Xpert testing with Simultaneous TPT if the goal is to increase TB treatment coverage and advance TB prevention. Our results also highlight implementation considerations related to the primacy of care cascades for achieving HIV treatment and TB control goals. Attrition along cascades (particularly related to treatment initiation and TPT continuation) undermine treatment and prevention opportunities from any algorithm.

As South Africa introduces Universal Xpert testing for PLHIV, our results suggest that decisions about the timing of TPT initiation could majorly determine effective TPT delivery and TB prevention. Delaying TPT until Xpert results are available prevents patients from benefiting from TPT during the early ART period when the risk of developing TB remains highest. Delaying TPT is also likely to result in lower TPT delivery overall, because TPT initiation is integrated into HIV care workflows around initial assessment and ART initiation. The latter effect might be mitigated with system changes promoting TPT initiation at follow-up visits and identifying patient-centered approaches to assure that those visits occur promptly once negative Xpert results are available. Simultaneous TPT initiation may also be less complex and more feasible than achieving high levels of uptake of delayed TPT.

When we set our model parameters to reflect current, sub-optimal programmatic follow-up and retention (estimated 25% of ART initiators not completing their first follow-up visit within three months, as well as approximately 3.5% discontinuing ART and 7% discontinuing TPT each month thereafter), pairing Universal Xpert with Simultaneous TPT could prevent only a small fraction of total TB incidence in the cohort (<10% in most simulations). The potential benefits of TPT (and ART) were thus reduced by loss from the care cascade. Additionally, because of large preventive effects of ART on TB incidence, much of the cohort’s incident TB occurred among those patients who disengaged from care. Thus, any effort to improve outcomes through TPT must include attention to both initiation, adherence to, and completion of TPT and adherence to and continued retention on ART.

Although Universal Xpert testing and Simultaneous TPT could increase both TB treatment and TPT coverage, some potential drawbacks exist. First, even with Universal Xpert testing, we projected that roughly 50% of patients with prevalent TB would remain untreated. This was due largely to the imperfect sensitivity of Xpert Ultra among an HIV-positive, partially asymptomatic patient population.[26] Second, Universal Xpert testing led to TB treatment of individuals whose sputum, if evaluated by culture, would have been culture-negative. Some of these Xpert-positive, culture-negative recipients may have true TB disease (including extrapulmonary forms) and thus benefit from treatment, but to the extent that such Xpert results are false-positive, the benefits of Universal Xpert must be weighed against the harms and costs of inappropriate treatment. Stronger evidence to distinguish true disease among those with trace Ultra results could reduce this. Finally, a drawback of early TPT initiation is that a larger proportion of patients with active TB will initiate TPT, raising concerns about acquired drug resistance. Mitigating this risk are (1)most such patients receive at most one month of therapy before switching to 4-drug treatment; (2)those who receive more than one month will typically be those with Xpert-negative TB, whose low bacterial burdens limit the risk of resistance acquisition (3)preventing more active TB reduces the opportunity for the emergence of resistance.[27] The growing use of two-drug TPT (3HP) regimens may further mitigate resistance-related risks. In addition, given the increased TPT completion observed with shorter regimens, switching to 3HP may also enhance the incidence impact of universal TPT initiation.

As with any modeling exercise, our results come with several limitations. First, data were limited for several model parameters, particularly those related to care continua. Recognizing that standard practice is continually changing and collection of detailed data about care practices may affect those practices, we used data from recent pragmatic studies where interventions were unlikely to influence outcomes of interest for this modeling. Second, we modeled universal initiation of TB screening under all algorithms; if clinicians were more likely to initiate a TB screening process based on symptom screening or laboratory testing, then more widely adopted approaches may lead to greater TPT uptake. Third, in modeling TB treatments, we focused on ART initiation and first follow-up visits. Some patients with TB may be diagnosed later or elsewhere, and thus long-term differences in TB treatment between algorithms may be smaller than we estimated. Fourth, while we modelled the occurrence and timing of the second visit to be the same across algorithms, it is possible that attendance at a follow-up visit could be influenced by earlier clinical events (e.g., those who do not start TPT may be less likely to attend a follow-up visit); this could increase the clinical benefits of TB screening algorithms that encourage patients to remain in care. Finally, our analysis focuses on South Africa, where TB prevalence is high and TPT is reasonably integrated into HIV care. While our results likely will hold true for immunocompromised populations in other TB-endemic settings, estimates of the impact of Universal Xpert testing strategies in such settings would require adjustments to our parameters.

In summary, our model demonstrates that Universal Xpert can increase TB treatment initiation for PLHIV initiating ART, with a potential downside of increased unnecessary TB treatment due to false positive test results. Pairing Universal Xpert with Simultaneous TPT leads to earlier TPT initiation, higher TPT uptake, and greater TB prevention compared to either post-Xpert TPT initiation or current symptom-based screening practices. Socio-economic and structural interventions to improve care retention and medication adherence for ART, TB treatment, and TPT are needed to compliment changes to algorithms and treatments, thus maximizing their impact.

## Supplementary Material

Appendix

## Figures and Tables

**Table 1.** Simulated clinical outcomes of each screening algorithm

Outcome	Symptom Screening	CRP Screening	Universal Xpert with Delayed TPT	Universal Xpert with Simultaneous TPT
**Among patients with TB at ART initiation (N=500 [450–548])**				
Received anti-TB treatment within 60 days	26% (24%–28%) 130 [108–153]	28% (26%–31%) 140 [117–170]	53% (50%–56%) 265 [225–307]	53% (50%–56%) 265 [225–307]
Inappropriately remained on TPT >30 days	22% (17%–28%) 110 [77–153]	19% (14%–25%) 95 [63–137]	8.0% (5.3%–11%) 40 [24–60]	32% (29%–34%) 160 [131–186]
**Among patients without TB at ART initiation (N=4500 [4452–4550])**				
Initiated TPT within 60 days	41% (33%–49%) 1845 [1469–2229]	54% (47%–62%) 2430 [2092–2821]	23% (17%–31%) 1035 [757–1411]	88% (86%–90%) 3960 [3830–4095]
Remained on TPT >30 days	38% (31%–45%) 1710 [1380–2048]	48% (41%–55%) 2160 [2092–2503]	24% (17%–32%) 1080 [757–1456]	72% (69%–74%) 3240 [3072–3367]
Developed incident TB within 2 years	3.7% (3.4%–4.0%) 166 [151–182]	3.5% (3.3%–3.9%) 158 [147–177]	3.8% (3.5%–4.2%) 171 [156–191]	3.4% (3.1%–3.7%) 153 [138–168]
Inappropriately received anti-TB treatment	1.2% (0.94%–1.5%) 54 [42–68]	0.55% (0.42%–0.71%) 25 [19–32]	3.6% (3.0%–4.3%) 162 [134–196]	3.6% (3.0%–4.3%) 162 [134–196]

TB: Tuberculosis. TPT: TB preventive treatment. CRP: C reactive protein. All results are a median and interquartile range across 1000 simulations, with 5000 patients per simulation. Absolute numbers are shown underneath all percentages in square brackets.

**Table 2.** Clinical outcome comparisons between screening algorithms among 5000 patients entering HIV care

	Algorithms Compared			
Outcome Compared	Universal Xpert with Simultaneous TPT vs Symptom Screen	Universal Xpert with Simultaneous TPT vs CRP Screen	Universal Xpert with Simultaneous TPT vs Universal Xpert with Delayed TPT	Universal Xpert with Delayed TPT vs Symptom Screen
People with TB treated within 60 days	134 (118 to 150)	121 (105 to 137)	0 (−4 to 4)	135 (118 to 150)
People without TB receiving TPT within 60 days	2102 (1819 to 2379)	1512 (1214 to 1794)	2891 (2622 to 3148)	−771 (−889 to −668)
People without TB completing at least 6 months of TPT	1236 (1017 to 1475)	872 (653 to 1099)	1750 (1500 to 2002)	−496 (−584 to −415)
Averted incident TB cases (over 2 years)	17 (14 to 22)	10 (7 to 14)	23 (19 to 28)	−5 (−7 to −4)
Averted TB deaths	12 (9 to 15)	7 (5 to 9)	15 (12 to 19)	4 (−5 to −3)

TB: Tuberculosis. TPT: TB preventive treatment. CRP: C reactive protein. All results are a median and interquartile range across 1000 simulations, with 5000 patients per simulation managed with the specified algorithm.

**Table 3.** Cost-effectiveness comparison between screening algorithms.

Algorithm Comparison	Universal Xpert with Simultaneous TPT vs Symptom Screening	Universal Xpert with Simultaneous TPT vs CRP Screening	Universal Xpert with Simultaneous TPT vs Universal Xpert with Delayed TPT	Universal Xpert with Delayed TPT vs Symptom Screening
Incremental Cost per Additional Patient Treated within 60 days	$1,648 ($1,431–$1,898)	$1,807 ($1,546–$2,094)	NA ($12,362-NA)	$1,238 ($1,060–$1,460)
Incremental Cost per Additional TPT Administration within 6 months	$200 ($170–$243)	$270 ($216–$340)	$39 ($26–$54)	NA (NA-NA)
Incremental Cost per Incident TB case Prevented	$12,686 ($10,109–$15,553)	$21,618 ($15,708–$31,287)	$2,492 ($1,576–$3,514)	NA (NA-NA)
Incremental Cost per Death Averted	$19,029 ($15,164 –$23,329)	$32,427 ($23,562–$46,931)	$3,738 ($2,362 – $5,271)	NA (NA-NA)

NA indicates that the more expensive algorithm had no incremental benefit in at least 50% (for median of NA), at least 25% (for upper quartile of NA), or at least 75% (for lower quartile of NA) of simulations.

## Data Availability

All data and code used to reproduce the figures and results of this paper can be found in the following gituhub repository: https://github.com/ruchitab24/IPT_Model
