# Supplementary material for: Modeling the impact of universal TB molecular testing and timing of TB preventive treatment during ART initiation in South Africa
Source: AIDS. Author manuscript; Available in PMC 2024 Jan 11. (PMC10782927; doi:10.1097/QAD.0000000000003707)
Supplement: Appendix [file NIHMS1929220-supplement-Appendix.docx]

**Appendix for Modeling the impact of universal TB molecular testing and timing of TB preventive treatment during ART initiation in South Africa**

**Supplementary Table S1. Baseline Characteristic and Care Delivery Model Parameters**

| **Parameter description** | | **Value (sampled range)**^†^ | **Source** | |  |
| --- | --- | --- | --- | --- | --- |
| Proportion with CD4 Count: | |  | Lower bound: (1)  Upper bound: (2) | |  |
| <200 | | 0.31 (0.261-0.358) |  | |  |
| <100, given <200 | | 0.49 |  | |  |
| >350, given >200 | | 0.458 |  | |  |
| Prevalence of TB, by CD4 count: | |  | (3–5)** | |  |
| <100 | | 0.23 (0.16-0.30)* |  | |  |
| 100-200 | | 0.13 (0.09-0.17) * |  | |  |
| 200-350 | | 0.072 (0.050-0.094) * |  | |  |
| >350 | | 0.05 (0.035-0.065) * |  | |  |
| Proportion unable to produce an Xpert specimen, among those: | |  | (6) | |  |
| with TB | | 0.075 (0.06-0.09) |  | |  |
| without TB | | 0.075 (0.06-0.09) |  | |  |
| Sensitivity of Xpert Ultra | | 0.68 (0.57-0.76) | (5) | |  |
| Specificity of Xpert Ultra | | 0.96 (0.93-0.98) | (7) | |  |
| Proportion with symptoms, given: | |  | (5,8) | |  |
| true TB and Xpert-positive | | 0.85 (0.82-0.88) |  | |  |
| true TB and Xpert-negative | | 0.75 (0.7-0.8) |  | |  |
| true TB and no sputum | | 0.84 (0.8-0.87) |  | |  |
| No TB | | 0.58 (0.43-0.71) |  | |  |
| Sensitivity of CRP (>10 mg/L), if: | |  |  | |  |
| true TB and Xpert-positive | | 0.94 (0.88-0.98) | (9) | |  |
| true TB and Xpert-negative | | 0.84 (0.74-0.90) | (10) | |  |
| true TB and no sputum | | 0.89 (0.8-0.93) | (10) | |  |
| Specificity of CRP | | 0.74 (0.61-0.83) | (5) | |  |
| Probability of performing an Xpert confirmatory test following a positive symptom or CRP screen (P_XC_) | 0.57 (0.48-0.67) | | | (11) |  |
| Probability of initiating TPT at the initial visit, following a negative symptom or CRP screen (P_T1_) | 0.55 (0.30-0.82) | | | Lower bound- TEKO Study (Clinical Trial Identifier [NCT02119130](https://clinicaltrials.gov/ct2/show/NCT02119130))  Upper bound- Fedisa Study (NCT04466488) |  |
| Probability of universally deferring TPT (even if asymptomatic) in Universal Xpert with Delayed TPT algorithm (P_UC_) | 0.90 (0.85-0.95) | | | Assumption |  |
| Probability of universally prescribing TPT (even if symptomatic) in Universal Xpert with Simultaneous TPT algorithm (P_UD_) | 0.80 (0.75-0.85) | | | Assumption |  |
| Probability of returning for follow up within 90 days^***^ | Main Analysis: 1  Sensitivity Analysis: 0.75 (0.60-0.90) | | | Main Analysis: Assume all individuals return for a follow-up visit  Sensitivity Analysis: Upper bound and point estimate from (12); lower bound based on Fedisa Study (NCT04466488) |  |
| Probability of starting treatment at the follow-up visit if positive Xpert result (P_T2_) | 0.925% (0.90-0.95) | | | Derived from PITC data (13) |  |
| Relative probability of initiating TPT at follow-up if eligible, versus at initial visit after negative symptom/CRP screen (RR_T2_) | 0.5 (0-1) | | | Lower bound- Assumption  Upper bound- TEKO Study ([NCT02119130](https://clinicaltrials.gov/ct2/show/NCT02119130)) |  |
| Probability of resuming TPT if follow-up visit is after 31 days (P_31T_) | 0.75 (0.60-0.90) | | | Assumption |  |
| Monthly rate of TPT discontinuation (d_t_) ^§^ | 0.07 (0.04-0.10) | | | (14) |  |
| Monthly rate of ART discontinuation (d_a_) | 0.035 (0.01-0.05) | | | Upper bound (14)  Lower bound (14,15) |  |
| TB hazard ratio, current TPT vs no TPT | 0.5 (0.4-0.6)^‡^ | | | (11) |  |
| TB hazard ratio (Current TPT vs completed IPT) | | 0.695^‡^ | (11) | |  |

* Uncertainty in TB prevalence was modeled as correlated across CD4 strata, by drawing a single uncertainty multiplier between 0.7 and 1.3 and applying it to all four CD4-specific point estimates, rather than modeling uncertainty in each CD4 stratum independently.

** Published data from Kwa-Zulu Natal in the early 2010s (4) were adjusted for temporal trends and regional variation to estimate present-day, country-wide prevalence by CD4 stratum using data from current WHO. This was done by scaling the prevalence values from Kwa-Zulu Natal by the proportional difference in incidence between this region and all of South Africa at the time those data were collected (4) and reducing these prevalence by approximately 10% to account for changes in the national prevalence of TB over time (5).

*** For those who return within 90 days, time to follow up is sampled for each patient from an empiric distribution with a mean of 28 days and IQR 28-42 days (Fedisa Study, NCT04466488).

†Mode (lower bound, upper bound) of triangle distributions that parameter values were sampled from.

‡ To represent uncertainty, two multipliers were sampled from triangle (0.8,1,1.2). One was applied to all incidence rates (to represent uncertainty in TB incidence in absence of TPT) and the other to the TPT effect parameter (to represent uncertainty in TPT’s protective effects).

§Modeled as inclusive of ART discontinuation, by sampling ART discontinuation at rate d_a_ and then sampling additional TPT discontinuation at rate d_t_ – d_a_.

**Supplementary Table S2. Incidence Parameter Values**

| **Parameter label** | **Parameter description** | **Point estimate*** | **Source** |
| --- | --- | --- | --- |
| I_AE_ | Annual TB Incidence, on ART <6 months, by baseline CD4: |  | (16,17) |
|  | CD4 <100 | 0.0823 |  |
|  | CD4 100-200 | 0.0467 |  |
|  | CD4 200-350 | 0.0116 |  |
|  | CD4 >350 | 0.0096 |  |
| I_AL_ | Annual TB Incidence, on ART 6-24 months, by baseline CD4: |  | (16,17) |
|  | CD4 <100 | 0.011 |  |
|  | CD4 100-200 | 0.0098 |  |
|  | CD4 200-350 | 0.0095 |  |
|  | CD4 >350 | 0.0045 |  |
| RR_IE_ | TB hazard ratio, current TPT vs no TPT | 0.5 | (11) |
| RR_0_ | TB hazard ratio, not on ART vs on ART <6 month |  | (18) |
|  | CD4 <100 | 6.25 |  |
|  | CD4 100-200 | 6.25 |  |
|  | CD4 200-350 | 2.941 |  |
|  | CD4 >350 | 2.33 |  |
| RR_CI_ | TB hazard ratio (Current TPT vs completed IPT) | 0.695 | (11) |
| R_M_ | Ratio of mortality averted per incident case averted | 0.66 | (19) |

* To represent uncertainty, two multipliers were sampled from triangle (0.8,1,1.2). One was applied to all incidence rates (to represent uncertainty in TB incidence in absence of TPT) and the other to the TPT effect parameter RR_IE_ (to represent uncertainty in TPT’s protective effects).

**Supplementary Table S3. Stratified Incidence Rates**

| **Incidence Rate Parameter** | **Description** | **Calculation** | **CD4 <100**  **Value (TB Cases/Person-Time)** | **CD4 100-200**  **Value (TB Cases/Person-Time)** | **CD4 200-350**  **Value (TB Cases/Person-Time)** | **CD4 >350**  **Value (TB Cases/Person-Time)** |
| --- | --- | --- | --- | --- | --- | --- |
| AI | Incidence of TB on early ART and IPT | I_AE(CD4)_*RR_IE_ | 0.041245 | 0.023433 | 0.005767 | 0.0047815 |
| AC | Incidence of TB on early ART and completed IPT | I_AE(CD4)_*RR_CI_ | 0.07811 | 0.04453 | 0.0109865 | 0.009125 |
| LI | Incidence of TB on long term ART and IPT | I_AL(CD4)_*RR_IE_ | 0.0053655 | 0.0049275 | 0.00273385 | 0.00226665 |
| LC | Incidence of TB on long term ART and completed IPT | I_AL(CD4)_*RR_CI_ | 0.0074825 | 0.0068255 | 0.003796 | 0.0031536 |
| A | Incidence of TB on early ART alone | I_AE(CD4)_ | 0.08249 | 0.04672 | 0.0115705 | 0.0095995 |
| L | Incidence of TB on long-term ART alone | I_AL(CD4)_ | 0.0107675 | 0.0098185 | 0.005475 | 0.004526 |
| I | Incidence of TB on IPT alone | (I_AE(CD4)_*.5+ I_AL(CD4)*_.5)*RR_0(CD4)_*RR_IE_ | 0.14527 | 0.088695 | 0.0125195 | 0.0082125 |
| C | Incidence of TB on completed IPT alone | (I_AE(CD4)_*.5+ I_AL(CD4)*_.5)*RR_0(CD4)_*RR_CI_ | 0.20221 | 0.123005 | 0.0174105 | 0.0114245 |
| Null | Incidence of TB on neither ART nor IPT | (I_AE(CD4)_*.5+ I_AL(CD4)_*.5)*RR_0(CD4)_ | 0.290905 | 0.177025 | 0.025039 | 0.016425 |

**Supplemental Methods 1. Treatment Cost Estimation**

For individuals with baseline TB, we assumed a small proportion (2.1%) had multi-drug resistant (MDR) whose treatment was more costly (9701 USD).^(20,21)^ For incident TB cases that could be averted by TPT (i.e., those that might differ between algorithms), we assumed all cases to be drug-susceptible, assumed that only 61% were treated based on case detection ratios estimated by WHO, and added the cost of a single Xpert test for diagnosis.^(22)^

Unit costs were derived from existing literature which used empirical data collection methods. In all studies, recurrent and capital were collected using a mix of bottom up and top-down techniques.

Cost of Xpert: Cunnama et al., (2016) used bottom-up costing through direct observations of Xpert testis in 10 South African laboratories across four provinces, in a trial evaluating Xpert compared to microscopy.^(23)^ Top-down approaches were used for expenditures which could not be collected from direct observations.

Monthly IPT costs: Kim et al., (2018) estimated the cost of IPT empirically through directly observation using ingredients approach at six clinics in the Eastern Cape province of South Africa.^(24)^ Recurrent and fixed costs were collected, time -and-motion studies conducted and top down methods used to complement bottom-up costing approaches.

Cost of active DS-TB treatment: TB treatment costs were derived from articles by Pooran et al., (2013), Cunnama et al., (2016) and Vassall et al., (2017)^.(21,23,25)^ Costs by Pooran et al., (2013) were estimated empirically from the routine programme before the implementation Xpert whereas and Cunnama et al., (2016) and Vassall et al., (2017) costs were from the same study described in the costing of Xpert above^.(21,23,25)^ The average cost provide excludes the costs of screening which made part of the total treatment costs.

Monthly cost of ART: Meyer-Rath et al., (2019) conducted a systematic review of HIV treatment costs and provided the most up to date cost from empirical estimates in literature.(26)

CRP screening pe person was based on kit costs: ($1.50 each; Bio Lab Diagnostics) and $1000 in equipment costs as an up front cost.

| **Cost** | **Low & High** | **Notes** | **Source** |
| --- | --- | --- | --- |
| Cost of Xpert | $US16.9-$US33.5 | Low (bottom-up), High (Top-down costing) | (23) |
| Monthly cost of *IPT* | $US34.11 | 12-month course, high estimate | (24) |
| Cost of active DST TB treatment | *US130* | Cost of active TB treatment | (21,23,25) |
| Monthly cost of ART | $ 249.15 | Per patient year | (26) |

**Supplemental Methods 2. Reported Outcomes**

Cascades of care for each algorithm: the mean proportion, across 1000 simulations, of eligible patients (of patients with TB at baseline for treatment-related interventions, and of patients without TB for TPT-related interventions) who complete each step of the cascade.

Clinical outcomes for each algorithm:

- receipt of anti-TB treatment within 60 days, among patients with baseline TB
- Individuals with TB who remained on TPT >30 days, among patients with baseline TB
- development of incident TB within 2 years, among patients without baseline TB
- initiation of TPT within 60 days, among patients without baseline TB
- inappropriate receipt of anti-TB treatment, among patients without baseline TB

Algorithms compared (pairwise):

- “Universal Xpert with Simultaneous TPT” vs “Symptom screening”
- “Universal Xpert with Simultaneous TPT” vs “CRP screening”
- “Universal Xpert with Simultaneous TPT” vs “Universal Xpert with Delayed TPT”
- “Universal Xpert with Delayed TPT” vs “Symptom screening”

Clinical outcomes compared between algortihms:

- The number of people with TB treated within 60 days
- Averted incident TB cases (over 2 years)
- The number of people without TB who start TPT within 60 days
- Averted TB mortality

Cost-effectiveness outcomes compared between algorithm (measured as difference in cost divided by difference in clinical outcome for compared algorithms):

- Incremental cost per additional patient with TB treated within 60 days
- Incremental cost per additional TPT administration within 6 months
- Incremental cost per incident TB case prevented
- Incremental cost per death averted

**Supplemental Methods 3. Sensitivity Analyses**

We examined how key model outcomes depended on the values of individual parameters. The two key outcomes considered were (1) the difference in incident TB between Universal Xpert with Simultaneous TPT versus Delayed TPT (chosen because the goal of simultaneous TPT initiation is to avert incident TB), and (2) the difference in treatment initiation between Universal Xpert with Simultaneous TPT and Symptom Screening (chosen because Universal Xpert aims to improve treatment initiation). For each of these key outcomes, we considered the model parameters in Table S1 one at a time, comparing high versus low values of the current parameter of interest, while varying all other parameters according to their sampled uncertainty distributions. To do this, for each “current parameter of interest”, we compared the key outcome in the 200 simulations with the highest values for the current parameter of interest (the upper quintile among the original 1000 simulations) to the same key outcome in the 200 simulations with the lowest values for the same current parameter of interest (the lowest quintile among the original 1000 simulations) (Figure S1)

The following scenarios were also considered:

1. Reducing follow-up visit completion from 100% to a more typical 75% (Table S1),
2. Concentrating the risk of incident TB shortly after initiation of ART (by increasing the incidence rate three-fold during the first two months, and decreasing it thereafter to maintain a constant one-year cumulative incidence in absence of TPT; Table S5),
3. Assuming no TPT discontinuation once initiated.

**Supplementary Table S4. Simulated clinical outcomes of each screening algorithm with loss to follow up.**

| **Outcome** | **Symptom Screening** | **CRP Screening** | **Universal Xpert with Delayed TPT** | **Universal Xpert with Simultaneous TPT** |
| --- | --- | --- | --- | --- |
| **Among patients with TB at ART initiation (N=500 [450-548])** | | | | |
| Receipt of anti-TB treatment within 60 days | 18% (16%-20%)  90 [72-110] | 20% (18%-21%)  100 [81-115] | 37% (35%-39%)  185 [158-214] | 37% (35%-39%)  185 [158-214] |
| Individuals with TB who remained on TPT >30 day | 18% (15%-22%)  90 [68-121] | 15% (11%-19%)  75 [50-104] | 5.8% (4.0%-8.0%)  29 [18-44] | 46% (44%-48%)  230 [198-263] |
| **Among patients without TB at ART initiation (N=4500 [4452-4550])** | | | | |
| Initiation of TPT within 60 days | 36% (30%-42%)  1620 [1336-1911] | 51% (44%-57%)  2295 [1959-2594] | 17% (12%-22%)  765 [534-1001] | 87% (86%-89%)  3915 [3829-4050] |
| Individuals without TB who remained on TPT >30 days | 33% (28%-39%)  1485 [1247-1775] | 46% (39%-52%)  2070 [1736-2366] | 17% (13%-22%)  765 [579-1001] | 75% (73%-77%)  3375 [3250-3504] |
| Development of incident TB within 2 years | 5.8% (5.4% -6.3%)  261 [240-287] | 5.7% (5.3%-6.2%)  257 [236-282] | 5.9% (5.5%-6.4%)  266 [245-291] | 5.5% (5.1%-5.9%)  248 [227-268] |
| Inappropriate receipt of anti-TB treatment | 0.82% (0.62%-1.0%)  37 [28-46] | 0.38% (0.29%-0.49%)  17 [13-22] | 2.5% (2.1%-3.0%)  113 [93-137] | 2.5% (2.1%-3.0%)  113 [93-137] |

* All results are a median and interquartile range across 1000 simulations of each algorithm. Absolute values are shown in square brackets below percentages.

**Supplementary Table S5. Clinical outcome comparisons between screening algorithms with concentrated TB risk within the first 1-2 months of ART initiation.**

|  | **Algorithms Compared** | | | |
| --- | --- | --- | --- | --- |
| **Outcome Compared** | **Universal Xpert with Simultaneous TPT vs**  **Symptom Screening** | **Universal Xpert with Simultaneous TPT vs**  **CRP Screening** | **Universal Xpert with Simultaneous TPT**  **vs**  **Universal Xpert with Delayed TPT** | **Universal Xpert with Delayed TPT**  **Vs**  **Symptom Screening** |
| The number of people with TB treated within 60 days | 134 (118 to 152) | 121 (106 to 138) | 0 (-4 to 4) | 135 (117 to 151) |
| Averted incident TB cases (over 2 years) | -27 (-33 to -22) | -16 (-22 to -11) | -37 (-44 to -28) | 9 (7 to 12) |
| People without TB receiving TPT within 60 days | 2072 (1809 to 2330) | 1482 (1204 to 1756) | 2880 (2616 to 3126) | -781 (-910 to -673) |
| People without TB who complete at least 6 months of TPT | 1226 (1006 to 1445) | 848 (653 to 1068) | 1734 (1491 to 1992) | -500 (-601 to -421) |
| Averted TB Mortality | -18 (-22 to -14) | -11 (-14 to -7) | -25 (-29 to -19) | 6 (5 to 8) |

* All results are a median and interquartile range across 1000 simulations of each algorithm.

**Supplementary Table S6. Simulated clinical outcomes of each screening algorithm with no discontinuation of TPT and ART**

| **Outcome** | **Symptom Screening** | **CRP Screening** | **Universal Xpert with Delayed TPT** | **Universal Xpert with Simultaneous TPT** |
| --- | --- | --- | --- | --- |
| **Among patients with TB at ART initiation (N=500 [450-548])** | | | | |
| Received anti-TB treatment within 60 days | 26% (24%-28%) | 28% (26%-30%) | 53% (50%-56%) | 53% (50%-56%) |
| Inappropriately remained on TPT >30 days | 24% (18%-30%) | 20% (15%-27%) | 8.6% (5.7%-12%) | 33% (31%-36%) |
| **Among patients without TB at ART initiation (N=4500 [4452-4550])** | | | | |
| Initiated TPT within 60 days | 42% (35%-50%) | 56% (48%-62%) | 25% (17%-32%) | 88% (87%-90%) |
| Remained on TPT >30 days | 40% (33%-48%) | 50% (43%-57%) | 26% (18%-33%) | 74% (72%-77%) |
| Developed incident TB within 2 years | 2.3% (2.1%-2.4%) | 2.1% (2.0%-2.3%) | 2.3% (2.2%-2.5%) | 2.0% (1.9%-2.2%) |
| Inappropriately received anti-TB treatment | 1.2% (0.93%-1.4%) | 0.56% (0.43%-0.70%) | 3.6% (3.0%-4.3%) | 3.6% (3.0%-4.3%) |

**Supplementary Table S7. TB diagnostic and therapeutic costs, by screening algorithm**

| **Costs** | **Symptom Screening** | **CRP Screening** | **Universal Xpert with Delayed TPT** | **Universal Xpert with Simultaneous TPT** |
| --- | --- | --- | --- | --- |
| TPT Administration | $46,026 | $55,679 | $31,386 | $87,601 |
| Xpert Testing | $57,548 | $32,126 | $167,500 | $167,500 |
| Incident TB Treatment | $11,229 | $10,783 | $11,567 | $10,138 |
| Baseline TB Treatment | $64,860 | $71,241 | $135,704 | $135,403 |
| CRP Testing | $0 | $12,500 | $0 | $0 |
| Total TB Diagnostic and Treatment costs | $179,662 | $182,330 | $346,157 | $400,642 |

**Supplementary Table S8. Stability of results across multiple runs.**

| **Outcome** |  | **Symptom Screening** | **CRP Screening** | **Universal Xpert with Delayed TPT** | **Universal Xpert with Simultaneous TPT** |
| --- | --- | --- | --- | --- | --- |
|  | **Among patients with TB at ART initiation (N=500 [450-548])** | | | | |
| Receipt of anti-TB treatment within 60 days | Run 1 | 26% (23%-28%) | 28% (26%-29%) | 53% (50%-56%) | 53% (50%-56%) |
|  | Run 2 | 26% (24%-28%) | 29% (26%-31%) | 54% (51%-56%) | 54% (51%-56%) |
|  | Run 3 | 26% (24%-28%) | 29% (26%-31%) | 53% (50%-56%) | 53% (50%-56%) |
|  | Run 4 | 26% (24%-28%) | 28% (26%-31%) | 53% (50%-56%) | 53% (50%-56%) |
|  | Run 5 | 26% (24%-28%) | 28% (26%-31%) | 53% (50%-56%) | 53% (50%-56%) |
| Individuals with TB who remained on TPT >30 day | Run 1 | 23% (18%-28%) | 19% (14%-25%) | 8.1% (5.4%-11%) | 32% (29%-34%) |
|  | Run 2 | 23% (18%-29%) | 20% (15%-25%) | 8.2% (5.7%-11%) | 31% (29%-34%) |
|  | Run 3 | 23% (18%-29%) | 20% (15%-25%) | 8.3% (5.8%-11%) | 32% (29%-34%) |
|  | Run 4 | 23% (18%-29%) | 20% (15%-25%) | 8.3% (5.6%-11%) | 32% (30%-34%) |
|  | Run 5 | 22% (17%-28%) | 19% (14%-25%) | 8.0% (5.3%-11%) | 32% (29%-34%) |
|  | **Among patients without TB at ART initiation (N=4500 [4452-4550])** | | | | |
| Initiation of TPT within 60 days | Run 1 | 42% (35%-49%) | 55% (48%-62%) | 24% (17%-31%) | 88% (87%-90%) |
|  | Run 2 | 41% (35%-50%) | 55% (48%-62%) | 24% (18%-32%) | 88% (87%-90%) |
|  | Run 3 | 42% (35%-50%) | 56% (48%-62%) | 24% (17%-32%) | 88% (87%-90%) |
|  | Run 4 | 42% (35%-50%) | 56% (48%-62%) | 24% (17%-32%) | 88% (87%-90%) |
|  | Run 5 | 41% (33%-49%) | 54% (47%-62%) | 23% (17%-31%) | 88% (86%-90%) |
| Individuals without TB who remained on TPT >30 days | Run 1 | 38% (32%-46%) | 48% (42%-55%) | 24% (17%-32%) | 72% (69%-74%) |
|  | Run 2 | 38% (32%-46%) | 48% (42%-55%) | 24% (18%-32%) | 72% (69%-74%) |
|  | Run 3 | 39% (32%-46%) | 49% (42%-55%) | 25% (18%-32%) | 72% (69%-74%) |
|  | Run 4 | 39% (32%-46%) | 49% (42%-55%) | 25% (18%-32%) | 72% (69%-74%) |
|  | Run 5 | 38% (31%-45%) | 48% (41%-55%) | 24% (17%-32%) | 72% (69%-74%) |
| Development of incident TB within 2 years | Run 1 | 3.7% (3.4%-4.1%) | 3.6% (3.3%-3.9%) | 3.9% (3.5%-4.2%) | 3.4% (3.1%-3.7%) |
|  | Run 2 | 3.7% (3.4%-4.1%) | 3.6% (3.3%-3.9%) | 3.9% (3.5%-4.2%) | 3.4% (3.1%-3.7%) |
|  | Run 3 | 3.7% (3.4%-4.1%) | 3.6% (3.3%-3.9%) | 3.9% (3.5%-4.2%) | 3.4% (3.1%-3.7%) |
|  | Run 4 | 3.7% (3.4%-4.1%) | 3.6% (3.3%-4.0%) | 3.9% (3.5%-4.2%) | 3.4% (3.1%-3.7%) |
|  | Run 5 | 3.7% (3.4%-4.0%) | 3.5% (3.3%-3.9%) | 3.8% (3.5%-4.2%) | 3.4% (3.1%-3.7%) |
| Inappropriate receipt of anti-TB treatment | Run 1 | 1.2% (0.93%-1.4%) | 0.55% (0.42%-0.72%) | 3.6% (3.0%-4.3%) | 3.6% (3.0%-4.3%) |
|  | Run 2 | 1.2% (0.96%-1.5%) | 0.57% (0.43%-0.72%) | 3.6% (3.0%-4.4%) | 3.6% (3.0%-4.4%) |
|  | Run 3 | 1.2% (0.96%-1.5%) | 0.55% (0.43%-0.71%) | 3.7% (3.0%-4.4%) | 3.6% (3.0%-4.4%) |
|  | Run 4 | 1.2% (0.96%-1.5%) | 0.55% (0.43%-0.71%) | 3.7% (3.0%-4.4%) | 3.6% (3.0%-4.4%) |
|  | Run 5 | 1.2% (0.94%-1.5%) | 0.55% (0.42%-0.72%) | 3.6% (3.0%-4.3%) | 3.6% (3.0%-4.3%) |

**Supplementary Figure 1. Sensitivity analyses.** We report the difference in A) incident TB cases between the Universal Xpert with Simultaneous TPT algorithm and Universal Xpert with Delayed TPT algorithm and B) treatment initiation between the Universal Xpert with Simultaneous TPT algorithm and Symptom Screening algorithm. Yellow bars show the distribution of outcomes among simulations for which the specified parameter is in the lowest quintile of its sampled values, and dark orange bars show the distribution of outcomes among simulations for which the specified parameter is in the highest quintile of its sampled values. The distribution of outcomes across all parameter values are shown in light orange. All distributions are represented as boxplots, with the endpoints of the colored bars indicating the interquartile range (IQR) and the error bars indicate the highest and lowest values no more than 1.5*IQR from the ends of the bars. Parameters are ordered from lowest to highest partial rank correlation coefficient (PRCC).

**References**

1. Yapa HM, Kim HY, Petoumenos K, Post FA, Jiamsakul A, Neve JWD, et al. CD4+ T-Cell Count at Antiretroviral Therapy Initiation in the “Treat-All” Era in Rural South Africa: An Interrupted Time Series Analysis. Clinical Infectious Diseases: An Official Publication of the Infectious Diseases Society of America. 2022 Apr 4;74(8):1350.

2. CD4 – National Health Laboratory Service [Internet]. [cited 2023 Mar 21]. Available from: https://www.nhls.ac.za/priority-programmes/cd4/

3. Kendall EA, Hussain H, Kunkel A, Kubiak RW, Trajman A, Menzies R, et al. Isoniazid or rifampicin preventive therapy with and without screening for subclinical TB: a modeling analysis. BMC Med. 2021 Dec 14;19(1):315.

4. TB Statistics South Africa [Internet]. TBFacts. [cited 2023 Mar 21]. Available from: https://tbfacts.org/tb-statistics-south-africa/

5. Dhana A, Hamada Y, Kengne AP, Kerkhoff AD, Rangaka MX, Kredo T, et al. Tuberculosis screening among ambulatory people living with HIV: a systematic review and individual participant data meta-analysis. The Lancet Infectious Diseases. 2022 Apr 1;22(4):507–18.

6. Sabur NF, Esmail A, Brar MS, Dheda K. Diagnosing tuberculosis in hospitalized HIV-infected individuals who cannot produce sputum: is urine lipoarabinomannan testing the answer? BMC Infectious Diseases. 2017 Dec 28;17(1):803.

7. Zifodya JS, Kreniske JS, Schiller I, Kohli M, Dendukuri N, Schumacher SG, et al. Xpert Ultra versus Xpert MTB/RIF for pulmonary tuberculosis and rifampicin resistance in adults with presumptive pulmonary tuberculosis. Cochrane Database Syst Rev. 2021 Feb 22;2:CD009593.

8. Dorman SE, Schumacher SG, Alland D, Nabeta P, Armstrong DT, King B, et al. Xpert MTB/RIF Ultra for detection of Mycobacterium tuberculosis and rifampicin resistance: a prospective multicentre diagnostic accuracy study. Lancet Infect Dis. 2018 Jan;18(1):76–84.

9. Lawn SD, Kerkhoff AD, Vogt M, Wood R. Diagnostic and prognostic value of serum C-reactive protein for screening for HIV-associated tuberculosis. Int J Tuberc Lung Dis. 2013 May;17(5):636–43.

10. Yoon C, Semitala FC, Atuhumuza E, Katende J, Mwebe S, Asege L, et al. Point-of-care C-reactive protein-based tuberculosis screening for people living with HIV: a diagnostic accuracy study. Lancet Infect Dis. 2017 Dec;17(12):1285–92.

11. Rangaka MX, Wilkinson RJ, Boulle A, Glynn JR, Fielding K, van Cutsem G, et al. Isoniazid plus antiretroviral therapy to prevent tuberculosis: a randomised double-blind, placebo-controlled trial. Lancet. 2014 Aug 23;384(9944):682–90.

12. Gosset A, Protopopescu C, Larmarange J, Orne-Gliemann J, McGrath N, Pillay D, et al. Retention in Care Trajectories of HIV-Positive Individuals Participating in a Universal Test-and-Treat Program in Rural South Africa (ANRS 12249 TasP Trial). J Acquir Immune Defic Syndr. 2019 Apr 1;80(4):375–85.

13. Mshweshwe-Pakela N, Hansoti B, Mabuto T, Kerrigan D, Kubeka G, Hahn E, et al. Feasibility of implementing same-day antiretroviral therapy initiation during routine care in Ekurhuleni District, South Africa: Retention and viral load suppression. South Afr J HIV Med. 2020 Aug 20;21(1):1085.

14. Kendall EA, Durovni B, Martinson NA, Cavalacante S, Masonoke K, Saraceni V, et al. Adherence to tuberculosis preventive therapy measured by urine metabolite testing among people with HIV. AIDS. 2020 Jan 1;34(1):63–71.

15. Maskew M, Brennan AT, Venter WDF, Fox MP, Vezi L, Rosen S. Retention in care and viral suppression after same-day ART initiation: One-year outcomes of the SLATE I and II individually randomized clinical trials in South Africa. J Int AIDS Soc. 2021 Oct;24(10):e25825.

16. Van Rie A, Westreich D, Sanne I. Tuberculosis in patients receiving antiretroviral treatment: incidence, risk factors and prevention strategies. J Acquir Immune Defic Syndr. 2011 Apr;56(4):349–55.

17. MUPFUMI L, MOYO S, SHIN SS, WANG Q, ZETOLA N, MOLEBATSI K, et al. High Incidence of tuberculosis in the first year of antiretroviral therapy in the Botswana National ART programme between 2011 and 2015. AIDS. 2019 Dec 1;33(15):2415–22.

18. Suthar AB, Lawn SD, del Amo J, Getahun H, Dye C, Sculier D, et al. Antiretroviral therapy for prevention of tuberculosis in adults with HIV: a systematic review and meta-analysis. PLoS Med. 2012;9(7):e1001270.

19. Badje A, Moh R, Gabillard D, Guéhi C, Kabran M, Ntakpé JB, et al. Effect of isoniazid preventive therapy on risk of death in west African, HIV-infected adults with high CD4 cell counts: long-term follow-up of the Temprano ANRS 12136 trial. Lancet Glob Health. 2017 Nov;5(11):e1080–9.

20. Musa BM, Adamu AL, Galadanci NA, Zubayr B, Odoh CN, Aliyu MH. Trends in prevalence of multi drug resistant tuberculosis in sub-Saharan Africa: A systematic review and meta-analysis. PLOS ONE. 2017 Sep 25;12(9):e0185105.

21. Pooran A, Pieterson E, Davids M, Theron G, Dheda K. What is the cost of diagnosis and management of drug resistant tuberculosis in South Africa? PLoS One. 2013;8(1):e54587.

22. TB profile [Internet]. [cited 2023 Mar 22]. Available from: https://worldhealthorg.shinyapps.io/tb_profiles/?_inputs_&lan=%22EN%22

23. Cunnama L, Sinanovic E, Ramma L, Foster N, Berrie L, Stevens W, et al. Using Top-down and Bottom-up Costing Approaches in LMICs: The Case for Using Both to Assess the Incremental Costs of New Technologies at Scale. Health Econ. 2016 Feb;25 Suppl 1(Suppl Suppl 1):53–66.

24. Kim HY, Hanrahan CF, Martinson N, Golub JE, Dowdy DW. Cost-effectiveness of universal isoniazid preventive therapy among HIV-infected pregnant women in South Africa. Int J Tuberc Lung Dis. 2018 Dec 1;22(12):1435–42.

25. Vassall A, Siapka M, Foster N, Cunnama L, Ramma L, Fielding K, et al. Cost-effectiveness of Xpert MTB/RIF for tuberculosis diagnosis in South Africa: a real-world cost analysis and economic evaluation. The Lancet Global Health. 2017 Jul 1;5(7):e710–9.

26. Meyer-Rath G, Rensburg C van, Chiu C, Leuner R, Jamieson L, Cohen S. The per-patient costs of HIV services in South Africa: Systematic review and application in the South African HIV Investment Case. PLOS ONE. 2019 Feb 26;14(2):e0210497.
